# Selective retina therapy (SRT) in patients with therapy refractory persistent acute central serous chorioretinopathy (CSC): 3 months functional and morphological results

**DOI:** 10.1007/s00417-020-04999-9

**Published:** 2020-11-18

**Authors:** Maximilian Büttner, Benjamin Luger, Wasim Abou Moulig, Bernd Junker, Carsten Framme, Christina Jacobsen, Katharina Knoll, Amelie Pielen, on behalf of the SRT Study Group, Wasim Abou Moulig, Marita Awe-Krüger, Anke Beckmann, Reginald Birngruber, Jan Hendrik Brahms, Ralf Brinkmann, Maximilian Büttner, Pascal Buley, Lisa Danzmann, Nicolas Feltgen, Carsten Framme, Hans Hoerauf, Christina Sophie Jacobsen, Bernd Junker, Katja Kleemann, Katharina Knoll, Vanessa Kübler, Benjamin Luger, Yoko Miura, Ulrike Peters, Amelie Pielen, Johann Roider, Christopher Rosenstein, Eric Seifert, Nina-Antonia Striebe, Dirk Theisen-Kunde, Jan Tode, Ingo Roland Volkmann

**Affiliations:** University Eye Hospital, Medical School Hannover, Carl-Neuberg-Str.1, 30625 Hannover, Germany

**Keywords:** Central serous chorioretinopathy, Persistent acute disease, Selective retina treatment, Micropulse laser, Subretinal fluid, Fluorescein angiography, OCT

## Abstract

**Purpose:**

Central serous chorioretinopathy (CSC) is a disease presenting with detachment of the neurosensory retina and characteristic focal leakage on fluorescein angiography. The spontaneous remission rate is 84% within 6 months. In this study, the efficacy of selective retina therapy (SRT) was examined in patients with therapy refractory persistent acute CSC defined by symptoms for at least 6 months and persistent subretinal fluid (SRF) despite eplerenone therapy.

**Material and methods:**

This is a prospective, monocentric observational study in 17 eyes (16 patients, mean age 42 years, 2 female). SRT was performed with the approved R:GEN laser (Lutronic, South Korea), a micropulsed 527-nm Nd:YLF laser device, with a train of 30 pulses of 1.7 μs at 100-Hz repetition rate at the point of focal leakage determined by fluorescein angiography (FA) at baseline (BSL). Visits on BSL, week 4 (wk4), and week 12 (wk12) included best corrected visual acuity (BCVA, logMar), central retinal thickness (CRT) on spectral domain optical coherence tomography (SD-OCT), and FA. Statistical analysis was performed by pair-by-pair comparisons of multiple observations in each case with Bonferroni correction for multiple testing. (IBM SPSS Statistics 25®).

**Results:**

Mean CRT at BSL was 387.69 ± 110.4 μm. CRT significantly decreased by 106.31 μm in wk4 (95%-KI: 21.42–191.2; *p* = 0.01), by 133.63 μm in wk12 (95%-KI: 50.22–217.03; *p* = 0.001) and by 133.81 μm (95%-KI: 48.88–218.75; *p* = 0.001) compared to BSL. Treatment success defined as complete resolution of SRF occurred at wk4 in 7/17 eyes (35.3%) and at wk12 in 10/17 eyes (58.8%). Re-SRT was performed in 7/17 eyes (41.2%) after an average of 107.14 ± 96.59 days. Treatment success after Re-SRT was observed in 4/6 eyes (66.6%, 12 weeks after Re-SRT). Mean BCVA did not change significantly from BSL to any later timepoint after adjusting for multiple testing. Notably, eyes with treatment success showed better BCVA at all timepoints and gained more letters compared to failures.

**Conclusion:**

Single or repetitive SRT may be an effective and safe treatment in 2 of 3 patients suffering from acute persistent CSC after 6 months of symptoms or more. We observed complete resolution of SRF in around 60% of eyes 12 weeks after first SRT treatment and also 12 weeks after Re-SRT treatment in eyes with persistent or recurrent SRF. Results on the long-term course after SRT are still pending.

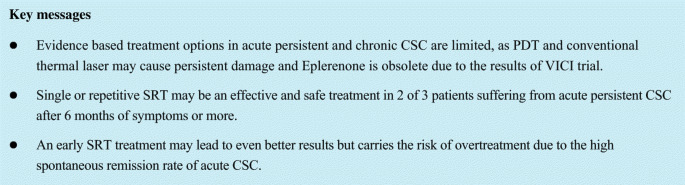

## Background

Central serous chorioretinopathy (CSC) is characterized by an idiopathic central exudative detachment of the neurosensory retina and affects mostly male patients aged 20 to 50. Patients experience visual disorders like metamorphopsia, decreased visual acuity, central scotoma, and low contrast sensitivity [1]. Fluorescein angiography (FA) shows single or multifocal spots of fluorescein leakage at the level of the retinal pigment epithelium (RPE) [2, 3]. Indocyanine green angiography (ICGA) shows choroidal hyperpermeability at the location of RPE leakage [4]. Nowadays, the principal diagnostic and monitoring device is spectral domain optical coherence tomography (SD-OCT) which shows the quantity of subretinal fluid (SRF) in acute cases. Modern SD-OCT has a sufficient resolution in order to discover small RPE detachments within the area of neurosensory detachment [5, 6]. SD-OCT also detects adjustments in chronic CSC, e.g., retinal thinning and cystoid retinal changes, corresponding with the depressed stage of visual acuity [7, 8].

Normally, CSC has a good outcome because SRF resolves spontaneously in up to 90% of patients within brief time periods, e.g., weeks to few months. Therefore, no treatment would be necessary, if restoration time is of no interest. Nonetheless, CSC can also become an acute persistent form for 6 months or more, so that patients suffer of notable persistent visual disturbances generated by repetitive or persistent events of acute CSC, or a chronic form with degenerative changes of RPE with or without persistent SRF [2, 4, 7, 13–15]. Besides “watchful waiting” for 3 months, a systemic therapy with eplerenone can be applied. Eplerenone 25 mg/day is given for 1 week and 50 mg/day for about 6 weeks after potassium lab control by the general practitioner [9]. In some studies, nearly one-third of patients showed a complete resolution after a median of 106 days under eplerenone treatment. The second third experienced a temporarily improvement while the remaining third of patients did not respond to or had to discontinue therapy [10]. Therefore, van Rijssen et al. suggested a systemic eplerenone therapy by the aforementioned regime as an option in highly symptomatic patients with disease activity for less than 2–4 months and/or in less symptomatic patients with persistent SRF for more than 4–6 months, if half-dose PDT is not available [11]. In 2020, the double-blind, randomized, placebo-controlled VICI trial showed no effect of oral eplerenone for patients with chronic CSC previously untreated for 4 months [12]. Our study was planned and conducted before the results of the VICI trial were published.

The definition of the subtypes of CSC is the topic of ongoing debate without a universally accepted classification system. The group of van Rijssen et al. defines acute CSC as “…acute-onset, dome-shaped serous detachment of the neuroretina, with spontaneous complete resolution of the resulting SRF in 3–6 months…” [11].

The same group described chronic CSC as “…serous detachment of the retina, with either small or more extensive areas of serous detachment of the RPE, together with atrophic changes to the outer retina and RPE developing secondary to choroidal vasculopathy… [with] …persistent serous detachment(s) on OCT for longer than 4–6 months” [11].

As our patients, by aforementioned definitions of forms of CSC by van Rijssen, showed “acute CSC”-like symptoms, but for a duration of at least 6 months without clearly typical RPE abnormalities as seen in chronic CSC, they do not really fit into either definition. That is why we described our patients as “acute persistent CSC” . A similar classification was proposed by Daruich et al., who described “non-resolving CSC” for cases without spontaneous revolving of SRF for 4–6 months but without atrophic RPE abnormalities characteristic for chronic CSC as a variant of acute CSC [31].

Regarding other therapeutic options focal laser photocoagulation with continuous wave lasers, argon (514 nm) or frequency-doubled Nd-YAG (532 nm) is thought to be a feasible therapeutic option if focal leakage is visible at FA [16–20]. However, conventional laser photocoagulation leads to thermal destruction of RPE and the neurosensory retina, small scotoma, and choroidal neovascularizations (CNV) can be generated [18, 19, 21]. That is why conventional laser photocoagulation is almost never performed if the RPE leakage is found close to the fovea.

Photodynamic therapy (PDT) is a feasible therapeutic option in chronic, active CSC with choroidal hyperpermeability, but not performed very often [4, 22].

Selective retina therapy (SRT) is a modern laser technology presented by Roider and colleagues initially using 5-μs argon laser pulses at 514 nm with a repetition rate of 500 Hz which selectively destroys the RPE cells with high peak temperatures around the melanosomes and leaves neurosensory retinal tissues unharmed [23–27]. SRT has already been carried out in patients with drusen due to age-related macular degeneration (AMD), with diabetic macular edema (DME) and patients with CSC [28]. SRT laser has been found to be safe and does not produce microscotoma in contrast to thermal laser. In addition, SRT laser does not produce CNV and the RPE can regenerate [29, 30]. Therefore, SRT appears to be the best available treatment option for acute, persistent acute, and chronic CSC, particularly if the RPE leakage is located close to the fovea. SRT presents the possibility of a safe treatment in patients with acute CSC and identifiable RPE leakage.

In our study, we examined the functional and morphological effects of SRT in patients with acute persistent CSC.

We did not perform SRT if the duration of reported symptoms was shorter than 6 months, to allow for a spontaneous resolution of SRF.

## Patients and methods

### Patients

We prospectively examined a set of patients with persistent acute CSC, defined clinically by symptom duration of 6 months or longer and previous treatment with eplerenone. All patients showed focal leakage on the level of the RPE on FA examination and substantial foveal subretinal fluid on SD-OCT but no signs of persistent damage due to chronic CSC (atrophy of the neurosensory retina and/or retinal pigment epithelium, secondary chorioretinal neovascularization). The indication for treatment of acute CSC with SRT was given when the RPE leakage was close to the fovea. Median distance of RPE leakage to the center of the fovea was 1100 μm (500–4300 μm), with 13/17 eyes showing RPE leakage within 1500-μm radius to fovea center, while 4/17 eyes showed RPE leakage at or above 2000-μm radius to the fovea center.

Patients were excluded, if the RPE leakage was outside of the vascular arcs. Those were treated with conventional laser. All patients gave their written informed consent before any study related procedures. The study conduct adhered to the tenets of the Declaration of Helsinki (ICH/ GCP) and was approved by the local ethics commitee (positive vote No. 7393 and No. 7243).

### Clinical examination

Every patient underwent a full eye examination, including best corrected visual acuity (BCVA, logMar), refraction, slit lamp biomicroscopy, and indirect ophthalmoscopy. FA and SD-OCT were performed in every patient using the Heidelberg Engineering Spectralis OCT (Heidelberg Engineering GmbH, Germany). The aforementioned examinations were repeated at scheduled study visits 4 weeks (wk4) and 12 weeks (wk12) after treatment, with patient-individual follow-up-visits after wk12. Retreatment (Re-SRT) was eligible at any study visit from wk4 onwards, if insufficient effect of the initial SRT (at BSL) was determined. After Re-SRT, the visits were performed as planned, with extra visits to follow-up Re-SRT where applicable. Due to individualized treatment regimen after week 12, the duration of follow-up varied significantly between individuals. To better illustrate the individual treatment regimen of patients within the study, please see Table 1. Treatment success was defined as complete resolution of SRF after Re-/SRT, treatment response was insufficient if SRF decreased but persisted after Re-/SRT, and treatment failure was defined as persistent or increased SRF despite SRT.

**Table 1:** Schedule of study visits

Patient no.	BSL/SRT	Wk4	Wk12	Re-SRT	Wk 4 after Re-SRT	Wk 12 after Re-SRT	Follow-up (days)
**#1**	x	x	x	Wk41	x	x	517
**#2**	x	x	x	Wk25	x	x	333
**#3**	x	x	x		n.a.	n.a.	180
**#4**	x	x	x		n.a.	n.a.	94
**#5**	x	x	x		n.a.	n.a.	192
**#6**	x	x	x		n.a.	n.a.	180
**#7**	x	x	x	Wk4	x	x	179
**#8**	x	x	x		n.a.	n.a.	82
**#9**	x	(x)	(x)		n.a.	n.a.	246
**#10**	x	x	x	Wk4	x	x	109
**#11**	x	x	x	Wk4	x	x	126
**#12**	x	x	x		n.a.	n.a.	126
**#13**	x	x	x		n.a.	n.a.	93
**#14**	x	x	x		n.a.	n.a.	85
**#15**	x	x	x	Wk17	x	x	174
**#16**	x		x	Wk12			91
**#17**	x	x	(x)		n.a.	n.a.	62

*x* visit attended, *(x)* visit attended but incomplete data or performed out of the planned visit window, *n.a.* not applicable, *empty box* study visit or treatment was not performed

### Treatment

The SRT laser (RGEN®, Lutronic, Korea, CE conformity according to 93/42 MDD) is a commercially not yet available original model, produced in conformity with essential requirements of the medical instrument directive 93/42EEC of the European Community (manufactured by Medical Laser Center Lübeck GmbH, Lübeck, Germany, in cooperation with Lumenis Ltd., Santa Clara, CA, USA) [32]. The system includes a frequency-doubled Q-switched Nd:YLF laser, which emits at a wavelength of 527 nm in a 100-Hz pulsed mode. Each pulse lasts 1.7 μs. Throughout every SRT exposition, a series of 30 pulses is applied [32]. In air, the laser spot size is 200 μm. SRT energy is titrated via an optoacoustic control during the application of every single spot to apply as much energy as is necessary to induce microbubble formation within the retinal pigment epithelium (RPE) but avoid persistent damage of the RPE and photoreceptors. We used a Mainster contact lens for single use which is connected to the main laser and detects optoacoustic signals during the procedure. The contact lenses and the laser itself were provided by Lutronic. Single pulse energies on the retina ranged from 90 to 170 μJ in treatment, 132.35 ± 22.23 μJ on average.

**Fig. 1:**
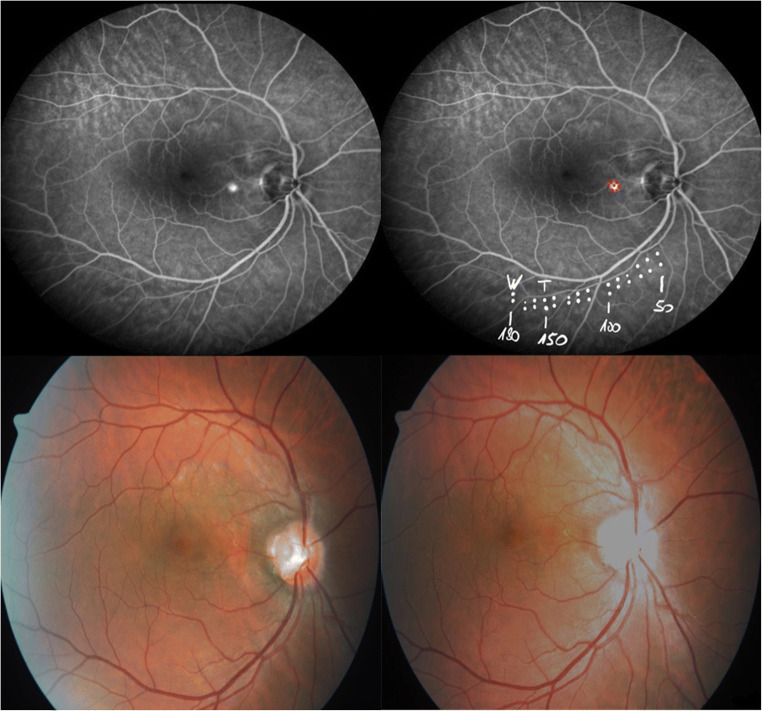
Upper left: fluorescein angiography with LP, upper right: planning of SRT treatment (red spots) and test spots on lower vascular arc (white spots) with marking of first visible laser effect (“W”) and energy chosen for treatment spots (“T”), lower left: fundus photography before SRT, and lower right: fundus photography after SRT (no visible laser spots)

The RPE lesions are ophthalmoscopically not visible during and after therapy [33, 34]. The SRT system is a slit lamp–adapted device that generates microbubbles forming around the intracellular melanosomes after the vaporization temperature at the melanosome’s exterior is outreached as mentioned above. Due to the microbubble dynamics and expansion, the cell collapses thermo-mechanically. Pressure waves released during formation of the bubbles and the collapse can be discovered by an ultrasonic transducer embedded into the contact lens. The signals are transformed by a PC [33]. Directly after every SRT exposition, the non-/existence of microbubbles is detected and shown on the screen of the laser device, this guarantees a direct control of a successful RPE damage [32]. Every patient within the study was treated by the same following regime: test exposures of different energy levels, starting at 50 μJ with subsequent increase by 10 μJ steps, were administered at the lower vascular arcade using TestRamp mode of RGEN device. The energy of the test spots was increased until the first spot could be seen ophthalmoscopically (median: 150 μJ, range 110–200 μJ). The maximum used energy was reduced by 20% in order to treat the actual CSC pathology with a minimum of 7 laser spots (center of the RPE leakage area plus a ring of a minimum of 6 laser spots around the center spot) depending on the lesion size at the site of RPE leakage as seen on FA (Fig. 1) using TestRamp mode.

Data was collected in a Microsoft Excel®–based table of the written reports of the clinical examinations and SD-OCT, FA, and FP images saved on server. Data was afterwards reviewed, completed, and prepared for data analysis using IBM SPSS Statistics 25®. Statistical analysis was performed by explorative data analysis, pair-by-pair comparisons of multiple observations with Bonferroni correction for multiple testing and non-parametric Friedman tests with Bonferroni correction for multiple testing. In the process, two eyes had to be partially excluded of testing due to missing data for CRT and visual acuity at different timepoints.

## Results

We treated 17 eyes of 16 patients with acute persistent CSC. Fourteen patients were male, and two female. The mean age of the patients was 42.2 years (± 7.9). Baseline BCVA (logMar) was 0.213 (± 0.196) and central retina thickness (CRT) 387.69 μm (± 110.4). All SRT treatments were performed without any significant adverse events, and no bleedings occurred at the site of the test or treatment spots, although a significant risk of bleeding is known. One hour after the SRT treatment, FA was performed to visualize the SRT-induced RPE damage due to progressive leakage. In every case, adequate treatment of the RPE leakage was ensured. Afterwards, no patient complained about central microscotoma.

We observed complete resolution of SRF in around 60% (10/17) of eyes 12 weeks after first SRT treatment and also 12 weeks after Re-SRT treatment (4/6) in eyes with persistent or recurrent SRF. We followed up 16 of 17 eyes (15 of 16 patients) after 4 weeks. In six of 17 eyes (35.3%), a complete resolution of subretinal fluid was seen on SD-OCT (treatment success). In 8 of the remaining 10 eyes (80%), the CRT was at least reduced compared to BSL without achieving complete resolution of SRF (insufficient treatment success). The mean visual acuity was 0.193 (± 0.183, *p* = 1.0) without significant difference to BSL, while the mean CRT decreased significantly by 106.31 μm (95%-KI: 21.42–191.2; *p* = 0.01).

At wk12, the mean BCVA changed to 0.12 ± 0.227 (95%-KI: − 0.021–0.207; *p* = 0.149) and the CRT decreased by 133.63 μm (95%-KI: 50.22–217.03; *p* = 0.001). Ten of 17 eyes showed a complete resolution of subretinal fluid (58.8% treatment success).

The last study visit was conducted after a median of 126 days (range: 62–517 days). Last study visit of one patient was performed early after 62 days due to data closure and personal reasons, while otherwise minimum was 82 days. The mean BCVA changed to 0.10 ± 0.24 (95%-KI: − 0.01–0.236; *p* = 0.081) without significant difference to BSL, while CRT decreased by 133.81 μm (95%-KI: 48.88–218.75; *p* = 0.001) in comparison to BSL. On the last study visit, 11 of 17 eyes (64.7% treatment success) showed a complete resolution of SRF, while 6 of 17 eyes still showed SRF (insufficient treatment effect or failure).

### Results by treatment success

The 11 eyes which finally achieved complete resolution of SRF (treatment success) started with a mean CRT of 383.09 μm ± 90.23 and changed significantly to 271.91 μm ± 51.78 (*p* = 0.003) at wk4, to 242.55 μm ± 31.89 (*p* = 0.01) at wk12, and to 240.36 μm ± 32.47 (*p* = 0.018) at last study visit. There was no significant difference between wk4, wk12, and last study visit in pair-by-pair comparisons. Mean BCVA was 0.14 ± 0.126 and changed to 0.12 ± 0.103 at wk4, 0.01 ± 0.129 at wk12, and 0.00 ± 0.126 at last study visit. Although there is statistical significant variance within the connected samples (*p* = 0.01), there is no significant difference between the 3 timepoints to BSL or between each other in pair-by-pair comparisons.

The 6 eyes without complete resorption at last study visit (treatment failures) started with a mean CRT of 397.8 μm ± 158.64 and changed to 302.2 μm ± 113.32 at wk4, to 279.4 μm ± 88.21 at wk12, and to 283.6 μm ± 84.71 at last study visit without significant difference in Friedman testing (*p* = 0.251). There was no significant difference in mean BCVA (*p* = 0.682) from BSL (0.36 ± 0.241) to wk4 (0.34 ± 0.23), wk12 (0.34 ± 0.23), or last study visit (0.30 ± 0.308).

**Fig. 2:**
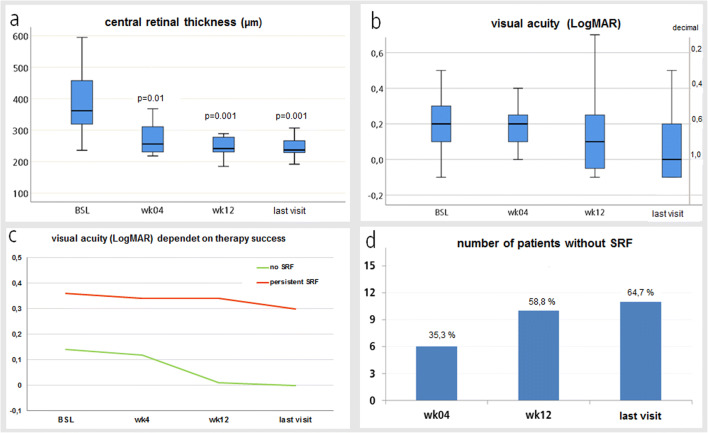
Elevated central retinal thickness (CRT) (**a**) and visual acuity (**b**) at 3 points in time compared to BSL. Visual acuity (**c**) dependent on therapy success (green = no subretinal fluid (SRF), *n* = 11; red = persistent SRF, *n* = 6). **d** Percentage of subjects without SRF after 4 weeks (wk), 12 wk., or at the last study visit (median: 126 days, range: 62–517 days)

**Fig. 3:**
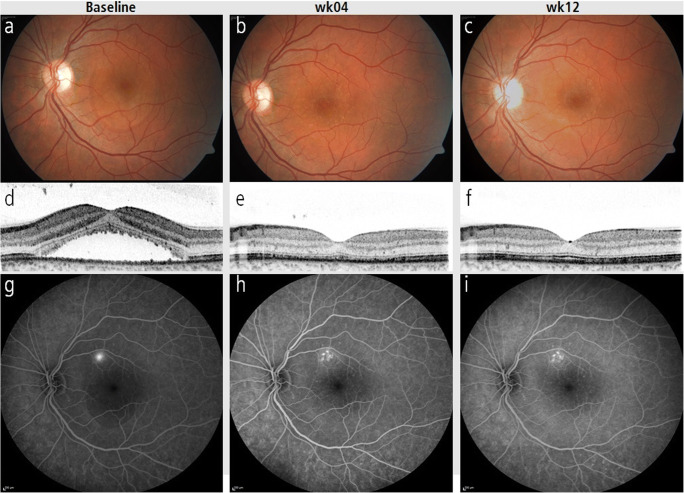
Subject 25. Fundus photography (**a**), SD-OCT (d), and fluorescein angiography (FA) late phase (**g**) with characteristic RPE leakage before selective retina therapy (SRT), after 4 weeks (**b**, **e**, **h**), and 12 weeks (**c**, **f**, **i**). SRT laser spots are only visible in FA (**h**, **i**), but not on ophthalmoscopy (**b**, **c**), test spots for dosimetry are visible near the lower vascular arcade

The results are displayed in Fig. 2, and a case report is shown in Fig. 3.

As the groups for successful and unsuccessful SRT treatments are very small, it is difficult to display statistical significant differences between the groups, especially adjusting for multiple testing, which is why we describe them. The results of the patients differentiated by therapy success are displayed in Table 2.

**Table 2:** schedule of study visits

	Treatment success	Treatment failure
Number of eyes	11/17	6/17
Median age in years (range)	39 (35–61)	40.5 (34–53)
Gender	2 female/9 male	6 male
LogMar BCVA at BSL (SD)	0.14 (± 0.126)	0.36 (± 0.241)
BCVA at last study visit	0.00 (± 0.126)	0.30 (± 0.308)
Mean CRT at BSL in μm (SD)	383.09 (± 90.23)	397.8 (± 158.64)
Mean CRT at last study visit in μm (SD)	240.36 (± 32.47)	283.6 (± 84.71)
Median distance RPE leakage–fovea center in μm (Range)	1100 (700–4300)	850 (500–2200)
Multiple-/diffuse RPE leakage in FA (%)	3/11 (27.3%)	2/6 (33.3%)
Median number of initial SRT laser spots (Range)	10 (6–22)	8 (6–13)
Mean initial SRT treatment energy in μJ (SD)	133.64 (± 21.86)	130 (± 20.82)
Re-SRT received (%)	3/11 (27.3%)	4/6 (66.7%)
Mean duration of follow-up in days (SD)	168.72 (± 123.25)	168.83 (± 81.18)

Two of 6 eyes considered as therapy failures at last study visit did not receive Re-SRT after insufficient initial SRT, while 4 of 6 eyes received Re-SRT in process. Two of those 4 eyes did not respond sufficiently neither to initial treatment nor to Re-SRT. One eye did not respond sufficiently to initial SRT and received Re-SRT but could not be followed up further due to data closure.

One patient (1 eye) showed complete resolution of SRF at wk12 after initial SRT, but developed SRF again during follow-up (174 days) and received Re-SRT. The patient showed complete resolution of SRF at wk12 after Re-SRT, but subsequently developed SRF again without complete resolution until last study visit.

Three patients, who were therapy successes at last study visit, received multiple SRTs in the process, which may explain apparently conflicting numbers.

Comparing eyes classified as therapy success to those classified as failure, the only significant difference we found was that eyes in therapy failures showed lower mean BCVA at BSL (0.36) than therapy successes (0.14). Failures showed persistent SRF and persistent worse BCVA compared to eyes with treatment success (see also Fig. 2c). There was no huge difference in change of CRT by micrometers at any timepoint.

All 6 therapy failures were eyes of male patients, although this is not surprising as only 2 patients of the whole collective were female. There was no difference in mean duration of follow-up.

The distance between RPE leakage and center of the fovea was a little lower with a median of 850 μm (500–2200 μm) in therapy failures compared to therapy successes with a median of 1100 μm (700–4300 μm).

Overall, 5 of 17 eyes showed multiple RPE leakages and/or diffuse leakage in larger areas in FA, with no huge difference between therapy failures (2/6) and therapy successes (3/11).

### Results of Re-SRT

In 7 eyes (41.2%) with insufficient effect, Re-SRT was performed after a median of 91 (range: 27–287) days with a mean energy of 131.67 ± 19.41 μJ. Mean CRT at Re-SRT was 382.5 μm ± 140.59, and mean LogMar BCVA was 0.083 ± 0.075.

We followed up on 6 eyes which received Re-SRT. Three of those 6 eyes had previously achieved complete resolution of SRF at wk12 after initial SRT but later developed SRF again. One case could not be followed up due to data closure. On week 4 after Re-SRT, mean CRT was 267.17 μm ± 101.66 without significant difference to BSL (*p* = 0.13); on week 12 after Re-SRT, mean CRT was 262.2 μm ± 84.79 (*p* = 0.063). There was no significant change in visual acuity 4 weeks and 12 weeks after Re-SRT.

Week 4 after Re-SRT, 2 of 6 eyes (33.3%) showed complete resolution of SRF and by week 12 after Re-SRT, 4 of 6 eyes (66.6%). Three of 6 eyes who received Re-SRT and were followed up still showed complete absence of SRF at last study visit. Two of those 3 eyes with permanent success of Re-SRT had shown complete resolution of SRF after initial SRT before. One of 6 eyes re-developed SRF later after showing no SRF 12 weeks after Re-SRT.

## Discussion

Our results show that SRT treatment can lead to a significant decrease of CRT accompanied by relevant visual improvement even after more than 6 months of disease in 2 of 3 eyes with acute persistent CSC (treatment success, defined as complete resolution of SRF).

CSC can occur as an acute persistent form, so that patients suffer of notable persistent visual disturbances often generated by repetitive events of acute CSC, and a chronic form with degenerative changes of RPE with or without persistent SRF [2, 4, 7, 13–15]. In our study, we recruited patients with CSC with persistent subretinal fluid (more than 6 months) in the foveal area after failure of therapy with eplerenone.

The rate of complete resolution of SRF after 12 weeks of 58.8% (treatment success) in this study is lower compared to a prospective randomized study by Klatt et al. (71.4%) [35]. However, the time of treatment in that study was much earlier (3 months after first occurrence of SRF compared to at least 6 months in our study). Therefore, some of the remissions might have occurred spontaneously, since 38% of the control group showed spontaneous remission [35]. In the aforementioned study, Re-SRT was not allowed, while in our study, Re-SRT could be performed, if initial SRT showed no sufficient effect at any time starting at wk4. Of 6 patients, who were treated with Re-SRT, 4 (66%) achieved complete resolution of SRF 12 weeks following Re-SRT. This indicates that SRT is effective even in those types of CSC that show recurrent episodes of SRF. The result is comparable to Framme et al., who achieved complete resolution of SRF in 5 of 6 cases with Re-SRT in patients with chronic recurrent CSC [29].

On the other hand, some of our cases may have already entered chronic disease state due to later treatment. In the present study, therapy failures showed a lower initial and final visual acuity than successfully treated eyes (Fig. 3(c)). Comparably, Framme et al. showed that in the case of chronic recurrent CSC 3 months after SRT, the absence of SRF is significantly lower at 19% than in cases of acute CSC at 100% [32].

Despite partly randomized studies with promising results [32, 35], SRT is still an off-label therapy. Inactivation of the RPE leakage point should be sought in the absence of spontaneous remission, since persistence can lead to chronification and secondary degeneration of RPE and photoreceptors [32]. An earlier treatment time (after 3 months) may lead to better results as shown by the above studies, but carries the risk of overtreatment due to the high spontaneous remission rate.

Photodynamic therapy (PDT) is seen as a feasible therapeutic option in chronic and acute persistent CSC (more than 4 months) with choroidal hyperpermeability, but leads to damages of the RPE and non-affected choriocapillary layer [4, 22]. We suggest that PDT should be considered, if treatment with SRT was not successful.

Eplerenone is the only pharmaceutical agent with a supposed direct pharmacological effect on CSC activity. But evidence from randomized, double-blind, placebo-controlled VICI trial showed no efficiency in CSC compared to placebo [12]. Therefore, eplerenone should no longer be recommended in patients with CSC. Results from previous studies showed that only 33% of the eyes treated with eplerenone achieved complete resolution of SRF after 6 weeks of therapy [9, 10]. Our results suggest a treatment success of SRT in 2 of 3 eyes in patients with acute persistent CSC. As SRT does no thermal damage to RPE, an early start of therapy, e.g., after 4–6 weeks with persistent SRF and significant reduction of vision, could be feasible, even at the risk of overtherapy [32, 35, 36]. The advantage of SRT at a later timepoint is the smaller number of potentially unnecessarily treated patients compared to earlier date of treatment. However, later treatment also may lead to a greater number of patients who already converted to chronic disease stage and therefore shows much worse results after SRT treatment. Based on our data, we suggest performing SRT at the earliest possible date after a considerable time of watchful waiting (of 3 months) to avoid conversion to chronic CSC. If there are already signs of chronic disease, SRT treatment could be tried first as non-damaging method compared to conventional laser or PDT, even if lower success rates may occur. Especially, SRT allows the treatment of LP at the fovea centralis, because no thermal damage to the RPE is applied. Furthermore Re-SRT is a reasonable option especially after successful initial SRT treatment with recurrent SRF at a later point of time, and also after unsuccessful initial SRT as shown in the data above. If complete resolution of SRF cannot be achieved by the aforementioned treatments, PDT should be considered.

## Conclusion

A successful treatment for longer disease duration of CSC has not been established so far. This study shows that SRT leads to treatment success of 64.7% in patients with acute persistent CSC and duration of disease activity of 6 months and more, with an additional 5.9% of at least temporary successful treatments. Results on the long-term course after SRT are still pending. Evidence-based treatment options in acute persistent and chronic CSC are very limited, eplerenone therapy is obsolete due to results of the VICI trial, and conventional thermal laser and PDT may cause persistent damage. SRT treatment in early stages of the CSC disease process may be reasonable, feasible, and safe to avoid conversion to chronic persistent CSC with its adverse effects of atrophy and secondary neovascularization.

## Abbreviations

AMD: age-related macular degeneration
CRT: central retina thickness
CSC: central serous chorioretinopathy
DME: diabetic macular edema
FA: fluorescein angiography
ICGA: Indocyanine green angiography
OCT: optical coherence tomography
PDT: photodynamic therapy
RPE: retinal pigment epithelium
LP: leakage point
SRF: subretinal fluid
SRT: selective retina therapy
